# 3D Conformal Radiotherapy Versus the Conventional Box Technique for Cervical Cancer: A Dosimetric Observational Study

**DOI:** 10.7759/cureus.89799

**Published:** 2025-08-11

**Authors:** Jagrati Yadav, Seema Gupta, Ajay K Venugopal, Arunima Ghosh, Indra J Gupta

**Affiliations:** 1 Department of Medical Oncology, Jawaharlal Nehru Medical College, Wardha, IND; 2 Department of Radiation Oncology, King George's Medical University, Lucknow, IND; 3 Department of Radiation Oncology, Era's Lucknow Medical College and Hospital, Lucknow, IND

**Keywords:** 3d-crt, cervical cancer, conventional radiotherapy, dosimetric evaluation, oar sparing

## Abstract

Background: Intensity-modulated radiotherapy (IMRT) and 3D-conformal radiotherapy (3D-CRT) have transformed radiation therapy in gynecologic cancers. However, access to advanced techniques in resource-limited settings is constrained. This study proposes a comparative dosimetric analysis using the cervical dosimetry mapping and planning (CERV-DOSIMAP) framework to evaluate the efficacy of 3D-CRT versus the conventional four-field box technique in locally advanced cervical cancer.

Methods: Radiotherapy planning was performed on 30 individuals with biopsy-confirmed International Federation of Gynaecology and Obstetrics (FIGO) stage IIB-IIIB cervical carcinoma using both conventional and 3D-CRT methods. Using CT-based organizing, volume goals (gross tumor volume (GTV), clinical target volume (CTV), and planning target volume (PTV)) and the organs at risk (OAR) were defined. Dose-volume histogram (DVH) data were analyzed. Key indices such as conformity index (CI), homogeneity index (HI), and dose coverage to PTV and OAR were statistically compared using paired t-tests.

Results: The 3D-CRT demonstrated significantly higher conformity (CI: 0.92 ± 0.03; p=0.001) and better homogeneity (HI: 0.08 ± 0.01; p=0.002) compared to conventional plans (CI: 0.85 ± 0.04; HI: 0.13 ± 0.02; p=0.003). There was a meaningful reduction in bladder, rectum, and femoral head doses with 3D-CRT.

Conclusion: The CERV-DOSIMAP framework validates 3D-CRT as a superior alternative to conventional techniques in improving target dose conformity while minimizing exposure to critical pelvic structures.

## Introduction

Cervical cancer (CC) remains a significant global public health concern, ranking as the fourth most common cancer in women worldwide, with over 662,000 new cases reported in 2022 alone [1]. The incidence and mortality rates are disproportionately higher in low- and middle-income countries due to limited access to preventive screening and advanced treatment modalities [2,3]. The high prevalence of human papillomavirus (HPV) types 16 and 18, found in approximately 70% of cervical cancer cases, has been widely acknowledged as the primary etiological factor [4].

Radiotherapy continues to play a pivotal role in the definitive and adjuvant treatment of locally advanced cervical cancer, especially when combined with chemotherapy [4-5]. Over the past two decades, technological advancements in radiotherapy, particularly three-dimensional conformal radiotherapy (3D-CRT) [6-7] and intensity-modulated radiotherapy (IMRT) [8-10], have revolutionized pelvic cancer treatment, offering the potential for improved tumor control while minimizing exposure to surrounding organs at risk (OAR) [11-13].

Unlike conventional four-field box techniques, which deliver radiation uniformly across wide anatomical areas, 3D-CRT and IMRT utilize anatomical imaging to shape radiation fields according to tumor geometry [14]. This not only enhances dose conformity to the planning target volume (PTV) but also significantly reduces radiation-induced toxicities to structures such as the bladder, rectum, and small intestine [15-17]. Studies have shown that optimized planning using advanced techniques improves local control, patient quality of life, and long-term survival outcomes in patients with cervical cancer [18-25]. However, despite its clinical benefits, the widespread use of IMRT is limited in resource-constrained settings due to complexity, longer planning times, and higher equipment demand [26-30]. In this context, 3D-CRT serves as a practical middle ground, offering better dosimetric precision than conventional techniques without the full resource requirements of IMRT.

The CERV-DOSIMAP framework presented a new and systematic method for the quantitative assessment and comparison of the dosimetric profiles of 3D-CRT with the conventional four-field box technique in the management of locally advanced cervical cancer. In contrast to prior research that provided fragmented or broad comparisons, the CERV-DOSIMAP framework facilitated an extensive mapping and strategizing of dose distribution over major pelvic structures. With a systematic dosimetric analysis, CERV-DOSIMAP provided higher accuracy in treatment assessment, with the potential to inform individualized radiotherapy planning and enhance therapeutic efficacy while reducing toxicity simultaneously. By focusing on dose-volume histogram (DVH) parameters, conformity and homogeneity indices, and OAR doses, this framework aims to establish a standardized, reproducible approach to optimize external beam radiotherapy planning in cervical malignancies.

## Materials and methods

Study design

The Institutional Ethics Committee of King George’s Medical University (Lucknow, UP, IND) approved this study (approval no. ECR/262/Inst/UP/2013/RR-19). The CERV DOSIMAP dosimetric study's organized workflow compares 3D-CRT and the traditional four-field box method for individuals with locally advanced cervical cancer. Every methodological step is described in the diagram, beginning with choosing patients based on International Federation of Gynaecology and Obstetrics (FIGO) staging standards and continuing with visualization as well as CT simulation methods for treatment planning. In compliance with International Commission on Radiation Units and Measurements (ICRU) guidelines, it emphasizes the delineation of gross and clinical as well as planning volume goals (gross tumor volume (GTV), clinical target volume (CTV), and planning target volume (PTV)) and details the inclusion of the most critical OAR. Two separate treatment plans were created, and dosimetric parameters such as D95, Dmean, conformity index (CI), and homogeneity index (HI) were evaluated. A clear and repeatable structure to guide comparative radiotherapy studies in the treatment of cervical cancer is provided by the statistical analysis, which used paired t-tests to determine importance.

Inclusion and exclusion criteria

Eligible participants were female patients aged 40 to 70 years who had histologically confirmed, locally advanced carcinoma of the cervix classified as FIGO stage IIB or IIIB and were scheduled for radical external‑beam chemoradiotherapy at our institution. Only cases with a baseline Eastern Cooperative Oncology Group performance status ≤ 2, no prior pelvic or abdominal radiotherapy, and no radiological or clinical evidence of distant metastasis at the time of staging work‑up were considered. Adequacy of imaging was ensured by requiring a contrast‑enhanced planning CT acquired with 3 mm slices that extended from the dome of the diaphragm to 5 cm below the ischial tuberosity, allowing complete target and organ‑at‑risk delineation. Patients had to consent to undergo two parallel treatment plans, one conventional four‑field box and one 3D‑CRT, prescribed to 45 Gy in 25 fractions, thereby permitting within‑subject dosimetric comparison.

Patients were excluded if they harbored para‑aortic or visceral metastases on staging imaging, were pregnant, had a history of hypersensitivity to iodinated contrast agents precluding contrast‑enhanced CT, or had severe renal, hepatic, or hematological dysfunction contraindicating concurrent cisplatin chemotherapy. Additional exclusion criteria were an inability to achieve reproducible bladder‑filling for CT simulation, inadequate immobilization, or incomplete digital dosimetry files that impeded dose-volume histogram extraction. Any case with missing contours for the prescribed organs at risk (bladder, rectum, sigmoid, bowel loops, or femoral heads) or without a verified physics quality‑assurance report was likewise excluded.

Radiotherapy procedure

All patients underwent CT-based simulation before treatment planning. Patients were positioned supine with arms raised and immobilized using a thermoplastic mask and leg support to ensure reproducibility. A full bladder protocol was followed, wherein patients were asked to empty their bladder and consume fluids before scanning. Contrast-enhanced planning CT scans with 3 mm slice thickness were acquired from the dome of the diaphragm to 5 cm below the ischial tuberosity. The acquired images were transferred to a treatment planning system (TPS) for contouring and plan generation. Two radiotherapy plans were created for each patient: plan A used the conventional four-field box technique (anteroposterior (AP)/posteroanterior (PA) and lateral fields), while plan B utilized a 3D-CRT approach with optimized beam angles based on individual anatomy. Both plans prescribed a total dose of 45 Gy delivered in 25 fractions over five weeks.

CT simulation

The CT simulations were performed for all patients using a consistent protocol to ensure planning accuracy and reproducibility. Patients were positioned in the supine position with a full bladder to displace bowel loops and reduce their exposure to radiation. Immobilization was achieved using a thermoplastic mask and leg support. Axial images were acquired using a multislice CT scanner with 3 mm slice thickness. The scan coverage extended from the dome of the diaphragm to 5 cm below the ischial tuberosity to encompass all relevant pelvic structures and lymphatic drainage regions. The datasets were then imported into the treatment planning system for further contouring and plan optimization.

Definition of target volumes

Target volumes were delineated according to the Radiation Therapy Oncology Group (RTOG) contouring guidelines. The GTV encompassed the cervix and any visible nodal involvement. The CTV included the uterus, parametrial tissue, upper third of the vagina, and regional lymphatics comprising the obturator, internal, external, and common iliac nodes. The PTV was generated by adding a uniform margin of 1 cm anteriorly and 0.5 cm in other directions around the CTV to account for setup uncertainties. The contoured OAR included the bladder, rectum, sigmoid colon, bowel loops, and femoral heads.

Treatment plan design

Each patient underwent radiotherapy planning using two distinct external beam radiotherapy techniques. Plan A involved the conventional four-field box technique, which utilized AP, PA, and lateral fields without any form of beam modulation. In contrast, plan B employed 3D-CRT, which incorporated patient-specific beam arrangements designed to improve dose conformity to the PTV while minimizing radiation exposure to the OAR.

Treatment planning for both techniques was performed on a commercial TPS. Each plan was prescribed a dose of 45 Gy in 25 fractions (1.8 Gy per fraction) over five weeks, consistent with standard radiotherapy protocols for cervical cancer. In 3D-CRT planning, four to six coplanar beams were used depending on anatomical variations, allowing better shaping of the dose around the PTV. The goal of optimization was to minimize the dose to the nearby OAR while attaining a minimum 95% dose cover of the PTV. Six MV photons of beam energy were employed, and dose restrictions were implemented per institutional procedures.

Assessment of the program

Quantitative dosimetric statistics gathered through DVH evaluation served as the foundation for evaluating each radiation treatment plan. The CI and the homogeneity index (HI), which evaluated how well the recommended dose adhered to and was evenly distributed within the PTV, respectively, were important measures of plan quality. The 3D-CRT strategies were compared to the conventional four-field box plans using the values of these indices, which were computed using standardized calculations. Furthermore, the assessment was based on volume restrictions as well as dose parameters such as D95, Dmean, and Dmax, OAR such as the rectum, bladder, and bowel, as well as femoral heads. The objective was to keep OAR dosages within suggested tolerance limits while guaranteeing at least 95% PTV coverage. Highly experienced radiation oncologists examined each plan to verify clinical acceptability and guarantee evaluation regularity.

Conformity index

The CI was used to assess how well the prescribed isodose conformed to the shape of the PTV. It was calculated using the formula CI = (VTref / VT​​) × (VTref / ​Vref), where VTref ​is the volume of PTV covered by the reference isodose, VT is the total PTV, and Vref is the volume of the reference isodose. A CI closer to 1 indicates better conformity.

Homogeneity index

The HI assessed how evenly the medication was distributed throughout the PTV. The calculation was as follows: HI = ​D5%​​ / D95%, where D5% is the dose received by 5% of the PTV (near-maximum dose), and D95% is the dose received by 95% of the PTV (near-minimum dose). An HI closer to 1 reflects better homogeneity.

Statistical analysis

The SPSS Statistics version 25.0 (IBM Corp., Armonk, NY, USA) was used for entering and analyzing all of the dosimetric data that was gathered, such as target volume coverage as well as OAR dose parameters. For the same group of clients, the means of constant variables were compared between the two radiotherapy modalities, i.e., the conventional four-field box planning and 3D-CRT, using paired t-tests. A p-value of less than 0.05 was considered statistically significant. Key parameters of D95, Dmean, CI, HI, and OAR metrics (e.g., V30, V40, Dmax) for both treatment plans were compared in the evaluation.

## Results

This dosimetric research retrospectively registered 30 female patients with histologically verified locally advanced cervical carcinoma (FIGO stage IIB IIIB). The median age of the patients was 54 years (±6.2 SD), with a range of 40 to 70 years. At the point of inclusion, none of the patients had a history of distant metastases or pelvic radiotherapy. All of the participants gave their informed consent in compliance with the institutional standards of ethics. Most patients received concurrent chemoradiation as per standard treatment protocol. The distribution of disease stages and treatment modalities is summarized in Table 1.

**Table 1:** Clinical characteristics of patients 3D-CRT: 3D-conformal radiotherapy, FIGO: International Federation of Gynaecology and Obstetrics

Characteristics		Number (total n = 30)	Percentage (%)
Age (years)	40-49	10	33.3
	50-59	12	40.0
	60-70	8	26.7
FIGO Stage	IIB	8	26.7
	IIIB	22	73.3
Concurrent chemotherapy		30	100.0
Treatment plan type	Plan A: Conventional four-field box	30	100.0
	Plan B: 3D-CRT	30	100.0

The majority of patients (12; 40 %) were aged 50 to 59 years, 10 (33.3 %) were in the 40 to 49 year group, and eight (26.7 %) were in the 60 to 70 year group. Concerning disease staging, 22 (73.3%) were diagnosed with FIGO stage IIIB carcinoma of the cervix, and eight (26.7%) presented with stage IIB disease. All patients received concurrent chemotherapy as part of their treatment protocol. For the purpose of this dosimetric comparison, each patient underwent two treatment planning strategies, namely plan A, which involved the conventional four-field box technique, and plan B, featuring 3D-CRT, to ensure a consistent basis for within-subject evaluation.

When the target volume dose distribution was compared, 3D-CRT outperformed the traditional four-field technique in terms of conformity and homogeneity (Table 2). Improved dose shaping near the PTV was indicated by the higher mean CI in 3D-CRT (0.92) as opposed to the traditional plan (0.85). Similarly, 3D-CRT had a better HI (0.08 vs. 0.13), indicating a more consistent dosage distribution inside the target. Better target coverage was also confirmed by the higher D95 values in 3D CRT, which represent the dose received by 95% of the PTV. The statistical significance of these differences was established at p < 0.005.

**Table 2:** PTV dose distribution characteristics CI: Conformity index; HI: Homogeneity index; D95: Dose to 95% of the PTV; PTV: Planning target volume; 3D‑CRT: Three‑dimensional conformal radiotherapy The p‑values were calculated using paired t‑tests.

Parameter	3D-CRT	Conventional plan	p-value
CI	0.92	0.85	0.001
HI	0.08	0.13	0.002
D95 (%)	96.2	92.5	0.003

The 3D-CRT's CI was substantially higher (0.92) than that of the traditional method (0.85), suggesting that the dose shipping more closely matched the target volume's form. A more consistent dose inside the PTV was also indicated by the lower HI in 3D-CRT (0.08 vs. 0.13). The D95 percentage, representing the dose received by 95% of the PTV, was also superior in the 3D-CRT group (96.2%) compared to the conventional group (92.5%). All differences were statistically significant with p-values < 0.005, confirming the dosimetric advantage of 3D-CRT in target coverage and precision.

The treatment lead time was notably reduced in the 3D-CRT group. The average machine occupancy time for 3D-CRT was approximately six minutes, compared to 10 minutes for the conventional box technique (Table 3). This represents a 40% reduction in overall treatment setup and delivery time, making 3D-CRT not only more precise but also more efficient in clinical workflow.

**Table 3:** Machine occupancy time 3D‑CRT: Three‑dimensional conformal radiotherapy

Technique	Machine occupancy time (minutes)	Time reduction (%)
3D-CRT	6	-
Conventional plan	10	40%

The 3D-CRT plans required an average of six minutes per treatment session, whereas the conventional four-field box technique took approximately 10 minutes. This indicates a 40% reduction in treatment time with 3D-CRT. The shorter machine occupancy time in 3D-CRT can be attributed to its more efficient and automated beam delivery process, eliminating the need for manual block adjustments typically required in conventional techniques. This time-saving advantage enhances treatment workflow and patient throughput in clinical practice.

Dosimetric analysis of the OAR showed that 3D-CRT significantly lowered mean doses to the bladder, rectum, and femoral heads compared to the conventional plan (Table 4). The mean dosage as well as the volume receiving ≥40 Gy (V40) for the bladder as well as rectum were significantly lower in the 3D-CRT group. With respective V40 readings of 72.3% and 87.8%, the bladder got an average dose of 42.1 Gy in 3D CT compared to 48.7 Gy in the traditional method. Similar reductions were noted for the rectum. These findings confirm that 3D-CRT provides superior organ sparing, reducing the potential for treatment-related toxicities.

**Table 4:** OAR dose comparison 3D‑CRT: Three‑dimensional conformal radiotherapy; OAR: Organs at risk; V40: Percentage of volume receiving at least 40 Gy The p‑value was obtained using the paired t‑test.

OAR	Mean dose for 3D-CRT (Gy)	Mean dose for conventional method (Gy)	V40 for 3D-CRT (%)	V40 for conventional method (%)	p-value
Bladder	42.1	48.7	72.3	87.8	0.001
Rectum	41.5	47.6	71.1	87.8	0.004
Femoral heads	37.8	42.3	-	-	0.006

**Figure 1:**
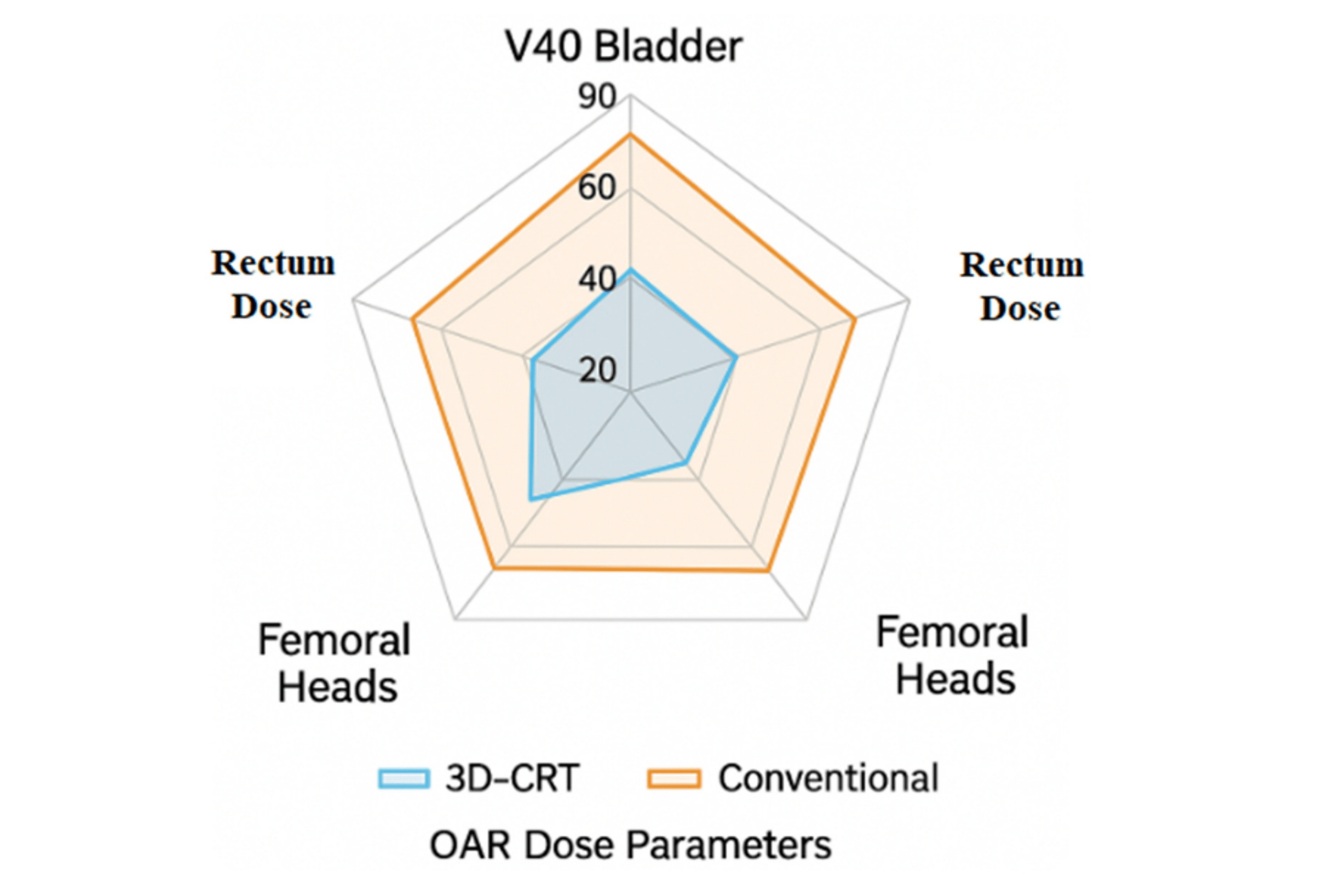
The OAR dose comparison OAR: Organs at risk; 3D‑CRT: Three‑dimensional conformal radiotherapy; V40: Percentage of volume receiving at least 40 Gy

Figure 1 (radar chart) compares the radiation dose received by the OAR between 3D-CRT and the conventional four-field box technique. The results demonstrate the dosimetric advantage of 3D-CRT in minimizing radiation exposure to critical pelvic structures.

## Discussion

The development of radiotherapy for cervical cancer has seen significant advancements in the past decade, best characterized by the transition from outdated box techniques to newer conformal techniques like 3D-CRT, IMRT, and volumetric modulated arc therapy (VMAT). All these modalities are designed to improve the therapeutic ratio by intensifying the target coverage and, at the same time, minimizing exposure of the OAR [16]. Dosimetric studies have consistently demonstrated that 3D-CRT offers superior spatial dose conformity in comparison to the conventional four-field techniques, with better accuracy in tumor targeting without additional toxicity to surrounding normal tissues [17-21].

The treatment of nodal disease is another pillar in cervical cancer radiotherapy. Extended field irradiation to para-aortic nodes, if required, requires accurate targeting to prevent undue renal and gastrointestinal toxicity. Present consensus lays great emphasis on the incorporation of imaging biomarkers and functional imaging in nodal delineation to ascertain accurate coverage [22]. The multimodality treatment with surgery and radiation, or even the omission of radiation in some high-risk cases altogether, is impending in the academic world, but long-term results are being investigated [23].

Emerging understanding of the host immune system and tumor microenvironment has fueled new areas of research. Besides physical dosimetry, hypoxia-induced radioresistance, immune modulation, and tumor heterogeneity are now influencing treatment response prediction and management [24]. Patient-centered radiotherapy paradigms like quality of life endpoints, patient-reported outcomes, and financial toxicity are increasingly being targeted besides oncologic efficacy [25].

There is renewed focus on radiotherapy delivery efficiency, particularly in high-volume oncology centers. Streamlining planning processes, automation during contouring, and hypofractionation regimens have been demonstrated to decrease machine occupancy time and enhance patient throughput without sacrificing outcomes [26]. Such efficiency gains are especially pertinent during pandemic and post-pandemic healthcare environments, where resource optimization is unavoidable.

Hypofractionation, though still in the investigation process for gynecologic malignancies, is increasingly being recommended as an acceptable alternative, especially in the palliative environment and some definitive contexts. Early-stage trials demonstrate equivalent local control rates with a reduction in hospital visits, potentially revolutionizing patient convenience and the economics of patient treatment [27]. In addition, the incorporation of artificial intelligence (AI) into radiotherapy planning and delivery holds much promise. The AI algorithms are capable of automating contouring, predicting toxicity, and determining the optimal beam configuration by analyzing anatomical and tumor data sets [28]. The incorporation of AI-driven tools into radiotherapy treatment plans is increasingly revolutionizing clinical practice. Machine learning algorithms are enhancing speed and accuracy through modalities like image segmentation and treatment verification [29]. More specifically, real-time imaging of internal anatomical structures by deep learning-based image guidance systems will reduce inter- and intra-fractional heterogeneity, a major problem in pelvic cancers like cervical cancer [30]. These technologies, however, require intense testing, ethical approval, and proper infrastructure to enable global uptake.

Our dosimetric analysis results, specifically the greater CI and HI and better sparing of the OAR in 3D-CRT compared to the traditional four-field technique, agree with trends reported in previous comparative dosimetry analyses. Algaoud et al. [16] also evidenced that 3D-CRT offered better conformity and dose homogeneity to pelvic targets in rectal cancer, with reduced high-dose irradiation to the OAR compared to previous techniques. These findings support our contention that 3D-CRT offers more geometric accuracy, which is critical in complex pelvic anatomy, as seen in cervical cancer. In addition, Octavia et al. [17] showed that 3D-CRT for cervical cancer yielded better D95 values and better homogeneity compared with conventional box planning, thus supporting the clinical significance of our findings.

Also, our evidence of lower mean doses and V40 exposure to the bladder, rectum, and femoral heads in our study is in line with the dosimetric advantage in IMRT and image-guided radiation therapy (IGRT) uses. Zeng et al. [18], in a large patient population, demonstrated that advanced conformal radiotherapy improved target coverage and minimized gastrointestinal toxicity due to improved OAR delineation and adaptive planning. Fang et al. [19] emphasized that conformal radiotherapy permitted dose escalation to nodal disease in cervical cancer with acceptable toxicity; this aligned with our evidence that increased conformity increases therapeutic margins without injuring surrounding anatomy. Further, the reduced treatment time evidenced in our application of 3D-CRT is supported by Ramiah and Mmereki [26], who reported that efficiency-improving technologies like 3D-based planning minimize machine occupancy and enhance workflow without diminishing clinical outcome.

However, certain limitations were noted. The relatively small sample size (n = 30) could limit the generalizability of the results to broader patient populations. The study did not assess correlations with clinical outcomes such as tumor control or survival, which could have strengthened the translational relevance of the dosimetric findings. Furthermore, the short duration of the study precluded any assessment of long-term toxicities or late adverse effects. Advanced radiotherapy techniques such as IMRT or VMAT were not included for comparative analysis. Lastly, the data were derived from a single-institutional cohort, which may reduce the external validity and applicability of the findings in diverse clinical environments.

## Conclusions

This study, through the application of the CERV-DOSIMAP framework, affirms the clinical and dosimetric superiority of 3D-CRT over the conventional four-field box technique in the treatment of locally advanced cervical cancer. The findings demonstrate that 3D-CRT provides enhanced conformity and homogeneity in dose distribution to the target volume while significantly reducing radiation exposure to organs at risk, including the bladder, rectum, and femoral heads. Additionally, 3D-CRT was associated with reduced machine occupancy time, improving treatment efficiency and patient throughput. These advantages are particularly meaningful for centers operating in resource-limited environments where advanced techniques like IMRT may not be feasible.

## References

[1] Pavelescu LA, Mititelu-Zafiu NL, Mindru DE, Vladareanu R, Curici A (2025). Molecular insights into HPV-driven cervical cancer: oncoproteins, immune evasion, and epigenetic modifications. Microorganisms.

[2] Shete K, Ghoulian J, Hu B, Alsyouf M (2024). Genitourinary cancer care in low-and middle-income countries: disparities in incidence and access to care. Soc Int Urol J.

[3] Tini P, Rubino G, Pastina P (2024). Challenges and opportunities in accessing surgery for glioblastoma in low-middle income countries: a narrative review. Cancers (Basel).

[4] Aimagambetova G, Bapayeva G, Ukybassova T, Kamzayeva N, Sakhipova G, Shanazarov N, Terzic M (2024). Risks of cervical cancer recurrence after fertility-sparing surgery and the role of human papillomavirus infection types. J Clin Med.

[5] Parisi S, Sciacca M, Ferrantelli G (2024). Locally advanced squamous cervical carcinoma (M0): management and emerging therapeutic options in the precision radiotherapy era. Jpn J Radiol.

[6] Patil DB, Zope MK, Singh D (2025). Dosimetric comparison of 3DCRT, IMRT, and Rapid Arc treatment techniques in cervical cancer: evaluating plan quality and organ at risk sparing. Mapan J Metrol Soc India.

[7] Hongo H, Tokuue K, Sakae T, Mase M, Omura M (2021). Robust treatment planning in intrafraction motion using Tomodirect™ intensity-modulated radiotherapy for breast cancer. In Vivo.

[8] Yamada T, Kawamura M, Oie Y (2024). The current state and future perspectives of radiotherapy for cervical cancer. J Obstet Gynaecol Res.

[9] Han F, Xue Y, Huang S (2024). Development and validation of an automated tomotherapy planning method for cervical cancer. Radiat Oncol.

[10] Jindakan S, Tharavichitkul E, Watcharawipha A, Nobnop W (2024). Improvement of treatment plan quality with modified fixed field volumetric modulated arc therapy in cervical cancer. J Appl Clin Med Phys.

[11] Peng C, Li X, Tang W (2024). Real-world outcomes of first-line maintenance therapy for recurrent or metastatic cervical cancer: a multi-center retrospective study. Int Immunopharmacol.

[12] Bhagyalakshmi AT, Velayudham R (2024). Assessing dosimetric advancements in spatially fractionated radiotherapy: from grids to lattices. Med Dosim.

[13] Pritanjali S, Rajhans K, Amrita R (2023). Dosimetric comparison of 3-dimensional conformal radiotherapy (3DCRT), intensity modulated radiotherapy (IMRT) and volumetric arc radiotherapy (VMAT) in cervical cancer treatment. Ann Radiat Ther Oncol.

[14] Singh M, Maitre P, Mody R, Murthy V (2024). Patterns of failure after prostate-only radiotherapy in high-risk prostate cancer: implications for refining pelvic nodal contouring guidelines. Clin Oncol (R Coll Radiol).

[15] Keita M, Xi C, Bah M (2025). Comparison of simplified intensity-modulated radiotherapy versus 3-dimensional conformal radiotherapy in locally advanced cervical cancer: a dosimetric study. Open J Obstet Gynecol.

[16] Algaoud MA, Al-Dhaibani N, Alomari AH (2024). Short course irradiation in rectal cancer: dosimetry study comparing 3D conformal radiotherapy versus intensity-modulated radiotherapy versus volumetric modulated arc therapy. J Radiat Res Appl Sci.

[17] Octavia AN, Devina RPS, Stevenly RJ, Sari AK (2025). The use of 3D-CRT technique in dose distribution analysis for cervical cancer treatment planning. J Inotera.

[18] Zeng Z, Wang W, Yan J, Liu D, Zhang F, Hu K (2024). Weekly image guidance in patients with cervical cancer treated with intensity-modulated radiation therapy: results of a large cohort study. Cancer Med.

[19] Fang C, Liu S, Xia J, Wu X, Zhu J, Ke G (2024). Clinical significance of intensity-modulated radiotherapy (IMRT) to the distant metastatic lymph nodes for metastatic cervical cancer. BMC Cancer.

[20] Mu Y, Wang H, Xu L (2024). Analysis of the therapeutic effect of synchronous integrated intensity modulated radiotherapy combined with chemotherapy in stage IIIc of cervical cancer. Front Oncol.

[21] Massobrio R, Bianco L, Campigotto B (2024). New frontiers in locally advanced cervical cancer treatment. J Clin Med.

[22] Cheung ES, Wu PY (2025). Current paradigm and future directions in the management of nodal disease in locally advanced cervical cancer. Cancers (Basel).

[23] Wang PH, Yang ST, Liu CH (2024). The selection of immune checkpoint inhibitors of programmed cell death (anti-PD-1) and its ligand (anti-PD-L1) makes matters more challenges for clinical practice. Taiwan J Obstet Gynecol.

[24] Klapp V, Paggetti J, Largeot A, Moussay E (2023). Targeting mRNA translation aberrations: a novel approach for therapy in chronic lymphocytic leukemia. Cancer Commun (Lond).

[25] Anghel B, Georgescu MT, Serboiu CS (2024). Optimizing palliative pelvic radiotherapy in gynecological cancers: a systematic review and analysis. Diagnostics (Basel).

[26] Ramiah D, Mmereki D (2024). Synthesizing efficiency tools in radiotherapy to increase patient flow: a comprehensive literature review. Clin Med Insights Oncol.

[27] Amjad R, Moldovan N, Raziee H, Leung E, D'Souza D, Mendez LC (2024). Hypofractionated radiotherapy in gynecologic malignancies-a peek into the upcoming evidence. Cancers (Basel).

[28] Cobanaj M, Corti C, Dee EC (2024). Artificial intelligence in the oncology workflow: applications, limitations, and future perspectives. Artificial Intelligence for Medicine.

[29] Kitson SL (2024). Modern Medical Imaging and Radiation Therapy. Cyber Secur Big Data AI Open Med Sci.

[30] Wáng YX, Zhao KX, Ma FZ, Xiao BH (2023). The contribution of T2 relaxation time to MRI-derived apparent diffusion coefficient (ADC) quantification and its potential clinical implications. Quant Imaging Med Surg.

